# Pathology in repeated transurethral resection of a bladder tumor as a risk factor for prognosis of high-risk non-muscle-invasive bladder cancer

**DOI:** 10.1371/journal.pone.0189354

**Published:** 2017-12-15

**Authors:** Bum Sik Tae, Chang Wook Jeong, Cheol Kwak, Hyeon Hoe Kim, Kyung Chul Moon, Ja Hyeon Ku

**Affiliations:** 1 Department of Urology, Korea University Ansan Hospital, Korea University College of Medicine, Ansan, Korea; 2 Department of Urology, Seoul National University Hospital, Seoul, Republic of Korea; 3 Department of Pathology, Seoul National University College of Medicine, Seoul, Korea; Johns Hopkins University, UNITED STATES

## Abstract

The prognostic value of repeat transurethral resection of bladder tumor (TURBT) in patients with diagnosed high-risk, non-muscle-invasive bladder cancer (NMIBC) was investigated. We retrospectively reviewed the medical records of patients treated from October 2004 to December 2013 at Seoul National University who underwent repeated TURBT within 2–6 weeks after an initial resection. The study enrolled patients who had been diagnosed with NMIBC at both the initial and repeat TURBT; patients with muscle-invasive tumors on repeat TURBT were excluded. We used stepwise multivariate Cox regression models stratified by study to assess the independent effects of the predictive factors and estimated hazard ratios (HRs) from the Cox models. We investigated a total of 198 patients who were diagnosed with high-risk NMIBC. In logistic regression analyses, number of bladder tumors (2–7: OR, 2.319; 8≤: OR, 3.353; p<0.05), initially high tumor grade (OR, 2.435; p = 0.040), and presence of carcinoma in situ lesion (OR, 3.639; p = 0.017) correlated with residual tumor in the repeated-TURBT specimen. T1 stage in repeated-TURBT significantly correlated with recurrence (HR, 1.837; p = 0.010) and progression (HR, 2.806; p = 0.029) in multivariate analysis. The high grades of tumors in repeated-TURBT also significantly correlated with progression but not recurrence in the multivariate analysis (HR 2.152; p = 0.008). In this study, the pathologic findings in repeated-TURBT correlated with recurrence and progression in high-risk NMIBC. Repeated-TURBT is valuable because it can predict the recurrence and progression of high-risk NMIBC in addition to obtaining accurate pathologic findings.

## Introduction

Approximately 80% of all newly diagnosed bladder cancers are non-muscle-invasive bladder cancer (NMIBCs) [1]. Transurethral resection of bladder tumor (TURBT) is the first-line treatment for NMIBC [2]. However, even in patients who have their bladder tumor completely removed with TURBT, up to 50% of these will have a recurrence of the cancer within 12 months.

The accurate diagnosis, staging, and subsequent prognostication of bladder cancer are achieved by TURBT, and repeated TURBT (re-TURBT) may change treatment strategy in high-risk NMIBC; this includes visibly recurring or residual tumors and muscular tissues around the initial TURBT scar, which can require random biopsy. When a muscle-infiltrating tumor is detected and upstaged at the re-TURBT, cystectomy or one of the bladder preservation protocols should be considered [3].

The benefit of re-TURBT is that it avoids staging errors as well as complete resection of residual tumor; in this sense, the re-TURBT may guide the surgeon toward a more appropriate therapeutic decision, which could potentially impact the prognosis and the curative rate of the technique [4]. A number of studies have presented that the rate of re-TURBT upstaging from T1 to T2 ranges from 0 to 28%, but one series found the rate to be up to 49% [5–10]. European Association of Urology (EAU) guidelines recommend that a repeat transurethral resection be performed within 2–6 weeks in patients with high-risk disease and in those with large or multiple tumors or with incomplete initial transurethral resections [3].

A number of studies have investigated the clinical value of re-TURBT, and most have focused on upstaging to muscle invasion bladder cancer or the benefits of restaging TURBT; however, few studies have investigated the meaning of re-TURBT pathology findings for patients with NMIBC. Thus, the objective of this study was to determine re-TURBT pathology as a prognosis risk factor in patients with a diagnosis of NMIBC.

## Materials and methods

The Institutional Review Board of Seoul National University Hospital approved this study (H-1611-096-809). Because we retrospectively performed our investigation, the IRB waived the need for informed consent documents from our patients. Patient information was anonymized and de-identified before we carried out the study, and we carried out all study procedures in accordance with the Declaration of Helsinki guidelines.

We retrospectively evaluated data collected from patients who underwent initial TURBT and re-TURBT between October 2004 and December 2013 at a single institution. All visual tumor findings were recorded on operation record sheets by the surgeon immediately after each operation, including tumor size, number, appearance, and location. Then, based on the EAU guideline, the finding of initial TURBT was indicated as re-TURBT, re-TURBT performed within 2–6 weeks after initial resection. At the re-TURBT, the bladder was reassessed to detect any residual tumors or missed lesions, and the previously resected sites and the peripheral areas around them were resected during the re-TURBT. All of the post-TURBT pathologic findings are reported using the 2004 World Health Organization (WHO) classifications. All histologic slides were reviewed by our pathologist, and we changed the previous recordings that used the 1973 WHO grade classifications (G1/G2/G3) to use the 2004 classifications (low grade/high grade).

In this study, we only analyzed patients who had been diagnosed with NMIBC at both the initial TURBT and the re-TURBT; therefore, we did not include patients who showed muscle-invasive tumors in re-TURBT. We also did not include patients for whom the surgeon documented ‘‘incomplete resection” or felt that biopsy alone was adequate after re-TURBT.

The point of our study was to evaluate the correlations between pathologic re-TURBT findings and recurrence or progression of high-risk NMIBC; we defined recurrence as detection and development of bladder cancer with any stage regardless of grade and progression as worsening of T stage to T2 during follow-up.

We used multivariate logistic regression analysis to assess the different risk factors for presence of residual tumors. Specifically, we used multivariable Cox regression analyses to calculate the hazard ratios for disease recurrence and progression, and we calculated the recurrence-free survival (RFS) and progression-free survival (PFS) curves using the Kaplan-Meier method and compared them with the log rank test. We analyzed the data using Statistical Package for Social Sciences, version 21.0 (SPSS, Chicago, IL, USA), obtaining the analytic statistics using the chi-square test or Fisher’s exact test; the differences were significant if P < 0.05.

## Result

### Study sample

A total of 206 patients were diagnosed with NMIBC after re-TURBT during the study period. We excluded eight patients we felt had had incomplete resections and/or biopsies only, leaving 198 patients who were suitable for analysis. Patient demographics and tumor characteristics are described in Table 1.

**Table 1:** Distribution of patients and tumor characteristics.

Variable		No. (%)
**Total patients included** **in analysis, N**		198
**Mean age, yr**		63.33 ± 11.07
**Gender**	Male	164 (82.8%)
	Female	34 (17.2%)
**Gross hematuria history**		147 (74.2%)
**Initial TURBT**		
**Tumor size**	Small to moderate (≤3cm)	142 (71.7%)
	Large (>3cm)	56 (28.3%)
**Number of tumors**	Single	92 (46.5%)
	2–7	82 (41.4%)
	7<	24 (12.1%)
**T stage**	CIS	7 (3.5%)
	Ta	30 (15.2%)
	T1	161 (81.3%)
**Tumor grade**	Low	27 (13.6%)
	High	171 (86.4%)
**Concomitant CIS**^**a**^		24 (12.1%)
**LVI**^**b**^		7 (3.5%)
**Muscle included**		81 (40.9%)
**Re-TURBT**^**c**^		
**T stage**	No tumor	91 (44.9%)
	Ta	68 (35.4%)
	T1	37 (18.7%)
	Other	2 (1.0%)
**Tumor grade**		
	No tumor	91 (44.9%)
	Low	26 (13.1%)
	High	81 (40.9%)
**Concomitant CIS**		31 (15.7%)

^a^CIS = Carcinoma in situ

^b^LVI = Lymphovascular invasion

^c^Re-TURBT = Repeated transurethral resection bladder tumor.

### Residual tumors in re-TURBT

Of the 198 patients who had been treated with re-TURBT, we found residual tumors in 107 (54.0%) of them. There were significant differences in baseline characteristics between the no residual tumor group and the residual tumor group concerning tumor grade, multiplicity and concomitant CIS (Table 2). Among these, the positive residual tumor rates were 63.1% (24/38) among the patients who had been diagnosed with initial TURBT stage Ta (or CIS) and 50.9% (85/167) among the patients who were initially diagnosed at T1 stage. Meanwhile, the positive rate among the low-graded patients was 33.3% (9/27), and that among the high-graded patients was 60.9% (99/161). In multivariate logistic regression analysis, number of bladder tumors (2–7: odds ratio [OR], 2.319; 7<: OR, 3.353; p<0.05), high tumor grade (OR, 2.435; p = 0.040), and presence of carcinoma in situ (CIS) lesion (OR, 3.639; p = 0.017) were revealed as independent risk factors for residual tumor in re-TURBT (Table 3). However, other pathologic findings including muscle included were not significant predictors of residual tumors.

**Table 2:** Residual tumor cases in re-TURBT according to initial pathology.

Parameter	No residual tumor (n = 91)	Residual tumor (n = 107)	P
**Gender (Female)**	13 (14.3%)	21 (19.6%)	0.211
**Age**			0.052
**<60**	34 (37.4%)	42 (39.3%)	
**61–70**	37 (40.7%)	28 (26.2%)	
**70<**	20 (22.0%)	37 (34.6%)	
**Previous UUT**^a^ **History**	7 (7.7%)	6 (5.6%)	0.379
**Prev. Recur History**	4 (4.4%)	10 (9.3%)	0.394
**T stage**			0.841
**Ta**	13 (14.3%)	17 (15.9%)	
**T1**	76 (83.5%)	85 (79.4%)	
**CIS**	2 (2.2%)	5 (4.7%)	
**Grade**			0.017
**Unknown**	0(0%)	0 (0%)	
**Low grade**	18 (19.8%)	9 (8.4%)	
**High grade**	73 (80.2%)	98 (91.6%)	
**Concomitant CIS**	5 (5.5%)	19 (17.8%)	0.007
**Size**			0.089
**≥3Cm**	21 (23.1%)	35 (32.7%)	
**Tumor Number**			0.007
**1**	53 (58.2%)	39 (31.2%)	
**2–7**	31 (34.1%)	51 (47.7%)	
**7<**	7 (7.7%)	17 (15.9%)	
**CRP**^b^	76.78±18.91	69.37±16.30	0.601

^a^UUT = Upper urinary tract tumor

^b^CRP = C-reactive protein

**Table 3:** Multivariate logistic regression analysis of residual tumor risk based on initial TURBT.

	Odd ratio (95% CI^a^)	P value
**LVI**^b^	2.265 (0.386–13.286)	0.365
**Gross hematuria**	1.904 (0.944–3.840)	0.072
**Number of tumor**		
**Single**	Ref	Ref
**2–7**	2.319 (1.230–4.372)	0.009
**7<**	3.353 (1.239–9.144)	0.018
**Tumor size >3Cm**	1.605 (0.827–3.113)	0.162
**High grade**	2.435 (1.043–5.683)	0.040
**Concomitant CIS**^c^	3.639 (1.261–10.506)	0.017
**T1 stage**	0.913 (0.363–2.299)	0.847
**Muscle included**	0.820 (0.441–1.524)	0.531

^a^CI = Confidence interval

^b^LVI = Lymphovascular invasion

^c^CIS = Carcinoma in situ

### Recurrence

**Fig 1:**
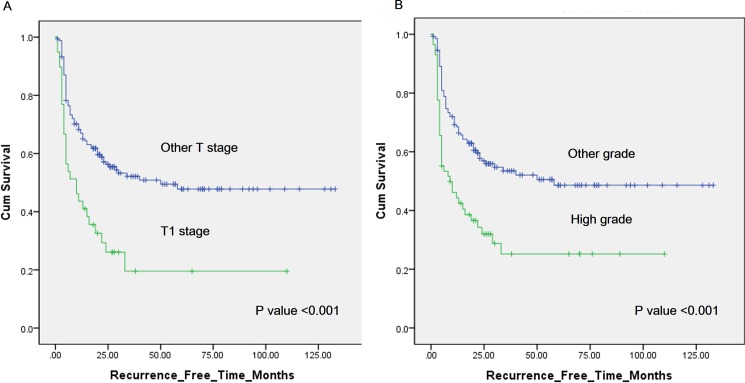
(A) Kaplan-Meier curves of recurrence-free survival (RFS) in patients with stage T1 (green) and other stages (blue) at the repeated transurethral resection of bladder tumor (re-TURBT). Two-year RFS was 26.1% and 56.0%, respectively, for the different stage categories (p<0.001). (B) Kaplan-Meier curves of RFS in patients with high tumor grades (green) and other grades (blue) at the re-TURBT. Two-year RFS was 32.0% and 56.9%, respectively, for the different grade categories (p<0.001).

A total of 103 patients showed tumor recurrence during follow-up. Univariate analysis revealed tumor number, lymphovascular invasion (LVI), residual tumor in re-TURBT, high grade in re-TURBT, and T1 stage in re-TURBT as significant predictive determinants of recurrence (Table 4); in addition, intravesical treatment was correlated with lower disease recurrence. Number of tumors (2–7; HR 2.047 p = 0.001, >7; HR 2.669 p = 0.001), LVI (HR 3.078 p = 0.017), residual tumors in re-TURBT (HR 1.786 p = 0.017), and T1 stage in re-TURBT (HR 1.837 p = 0.010) remained significant predictors of recurrence in multivariate analysis. Patients at stage T1 in re-TURBT showed a two-year recurrence-free survival rate of 26.1%, whereas patients in other stages had a rate of 56.0% (p<0.001; Fig 1). In addition, high grades in re-TURBT pathology also have lower RFS than do lower grades by Kaplan-Meier analysis (two-year, 32.0% vs 56.9%, p<0.001).

**Table 4:** Univariate and multivariate analyses of bladder cancer recurrence.

		Univariate		Multivariate	
		HR (95% CI)	P value	HR (95% CI)	P value
	Age	1.011 (0.991–1.031)	0.277	-	-
	Intravesical treatment	0.623 (0.416–0.931)	0.021	0.592 (0.391–0.895)	0.013
**Initial TURBT findings**	LVI	2.616 (1.062–6.440)	0.036	3.078 (1.222–7.749)	0.017
	Tumor Size >3Cm	0.936 (0.757–1.158)	0.544	-	-
	Tumor number				
	Single	Ref	Ref	Ref	Ref
	2–7	2.070 (1.104–3.880)	0.023	2.047 (1.324–3.165)	0.001
	>7	3.883 (1.375–10.969)	0.008	2.669 (1.504–4.738)	0.001
	T1 stage	1.153 (0.684–1.941)	0.593	-	-
	High grade	1.121 (0.638–1.972)	0.691	-	-
	Concomitant CIS	0.920 (0.492–1.721)	0.794	-	-
**Re-TURBT findings**	Residual tumor	2.230 (1.474–3.372)	<0.001	1.786 (1.111–2.871)	0.017
	T1 stage	2.387 (1.532–3.720)	<0.001	1.837 (1.154–2.924)	0.010
	Concomitant CIS	1.175 (0.697–1.980)	0.546	-	-
	High grade	2.212 (1.480–3.305)	<0.001	1.031 (0.519–2.040)	0.930

CIS = Carcinoma in situ, LVI = Lymphovascular invasion, Re-TURBT = Repeated transurethral resection bladder tumor. HR = Hazard ratio, CI = Confidence interval

### Progression

**Fig 2:**
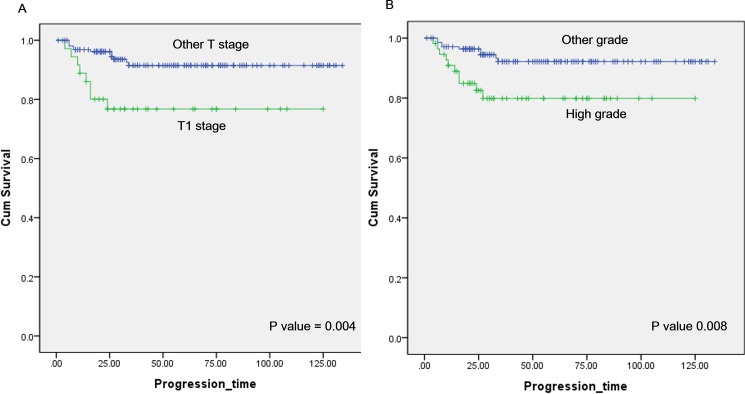
(A) Kaplan-Meier curves of progression-free survival (PFS) in patients with T1 stage (green) and other stage (blue) at the repeated transurethral resection of bladder tumor (re-TURBT). Two-year PFS was 76.8% and 94.4%, respectively, for the different stage categories (p = 0.004). (B) Kaplan-Meier curves of PFS in patients with high grade (green) and other grade (blue) at the re-TURBT. Two-year RFS was 82.5% and 94.5%, respectively, for the different grade categories (p = 0.008).

During follow-up, a total of 19 patients developed disease progression, all of whom had high-grade T1 tumors at the initial resection. Ten patients had high-grade T1 tumor after re-TURBT, and five had concomitant CIS after initial and re-TURBT. Eight patients received radical cystectomy, but 11 patients denied it or any further treatment. Among the patients who refused further treatment, six died during follow-up, and others did not visit our department; otherwise, all patients who received radical cystectomy in this analysis survived during our follow-up. Multivariate analysis revealed that stage T1 (HR 2.806 p = 0.029), high grade in re-TURBT (HR 2.152 p = 0.08), and age (HR 1.068 p = 0.014) were significant predictors of disease progression (Table 5). For patients who showed stage T1 in re-TURBT, the progression-free survival rate at two years was 76.8%, whereas the value for the patients in the other stages in re-TURBT was 94.4% (p = 0.004) (Fig 2). In addition, patients with high re-TURBT grades also had lower PFS than did patients with other grades in re-TURBT pathology based on Kaplan-Meier analysis (two-year, 82.5% vs 94.5%, p = 0.008).

**Table 5:** Univariate and multivariate analyses of bladder cancer progression.

		Univariate		Multivariate	
		HR (95% CI)	P value	HR (95% CI)	P value
	Age	1.078 (1.023–1.136)	0.005	1.068 (1.013–1.126)	0.014
	Intravesical treatment	0.516 (0.206–1.288)	0.156	-	-
**Initial TURBT findings**	LVI	5.833 (1.691–20.116)	0.005	0.614 (0.317–1.189)	0.148
	Tumor Size >3Cm	1.519 (0.598–3.836)	0.380	-	-
	Tumor Number				
	Single	Ref	Ref	Ref	Ref
	2–7	0.509 (0.253–1.026)	0.059	0.600 (0.291–1.238)	0.167
	>7	1.209 (0.662–2.208)	0.538	1.147 (0.606–2.170)	0.673
	T1 stage	3.914 (0.523–29.325)	0.184	-	-
	High grade	26.186 (0.118–579.691)	0.236	-	-
	Concomitant CIS	0.937 (0.216–4.060)	0.931	-	-
**Re-TURBT findings**	Residual tumor	1.588 (0.625–4.035)	0.331	-	-
	T1 stage	3.502 (1.408–8.712)	0.007	2.806 (1.111–7.084)	0.029
	Concomitant CIS	1.199 (0.348–4.124)	0.774	-	-
	High grade	3.952 (1.525–10.240)	0.005	2.152 (1.224–3.873)	0.008

CIS = Carcinoma in situ, LVI = Lymphovascular invasion, Re-TURBT = Repeated transurethral resection bladder tumor. HR = Hazard ratio, CI = Confidence interval

### Intravesical treatment

**Fig 3:**
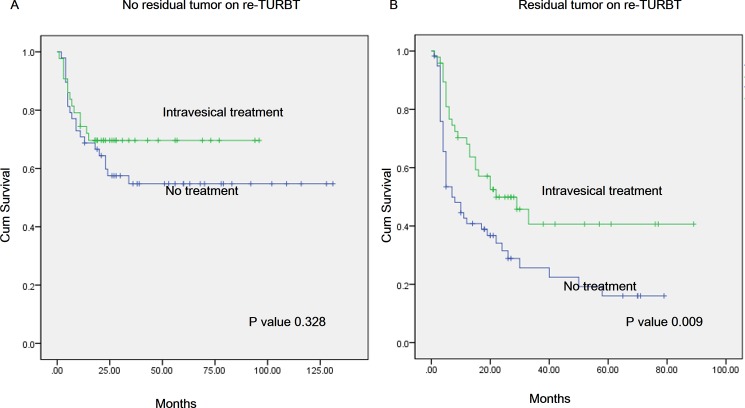
(A) Kaplan-Meier curves of progression-free survival (RFS) in patients with intravesical treatment patients (green) and no treatment patients (blue) in no residual tumor group after repeated transurethral resection of bladder tumor (re-TURBT). Two-year PFS was 69.6% and 57.5%, respectively, for the different treatment categories (p = 0.328). (B) Kaplan-Meier curves of PFS in patients with intravesical treatment patients (green) and no treatment patients (blue) in residual tumor group after re-TURBT. Two-year RFS was 41.7% and 31.5%, respectively, for the different treatment categories (p = 0.008).

For patients who showed residual tumor on re-TURBT (107 patients), 47.9% patients were received intravesical instillation treatment. Overall 2-year recurrence-free survival (RFS) rates in the no treatment group were poor compared to those in the intravesical treatment group (41.7% vs 31.5%, p = 0.009) (Fig 3B). However no residual tumor on re-TURBT patients (91 patients), 47.3% patients were received intravesical instillation treatment. Overall 2-year recurrence-free survival (RFS) rates in the no treatment group were relative poor compared to those in the intravesical treatment group but it was not statistically significant (57.5% vs 69.6%, p = 0.328) (Fig 3A).

## Discussion

Whereas most re-TURBT studies have focused on upgrading to muscle-invasive cancer, few have analyzed the clinical meaning of the pathologic findings of re-TURBT in high-risk NMIBC patients. For this reason, we wanted to understand the correlations between re-TURBT pathology and recurrence and prognosis in these patients.

The initial step in managing bladder cancer is complete resection of the transurethral bladder tumor. Some reports have found that the rates of residual bladder tumors perceived in re-TURBTs range from 33% to 76% [11], and our study showed a similar rate of residual tumors. A previous study reported that the factors that affected residual tumors in re-TURBT are tumor stage, grade, and size and interval between two TURBTs [12]. Among these, tumor size and stage did not significantly predict residual tumors in our study; however, multiplicity of tumors and concomitant CIS lesions were revealed as predictors of residual tumors.

Herr et al. published the largest repeat TURBT series, summarizing unpublished data on routine repeat TUR and including 1,312 patients with NMIBC [13]. They found residual disease in 51%–78% of patients, with the highest rate in the group with T1 disease at initial TUR. The natural history of the residual tumors after TURBT is not yet clear. However, a few studies have reported that the presence of residual tumors is a potential risk factor for disease recurrence and poor prognosis [6, 10, 14, 15]. Our study also showed that residual tumor findings after re-TURBT were associated with disease recurrence, although our results did not reach statistical significance for disease progression.

Grims et al. demonstrated that tumor stage and grade at initial TURBT predicted residual tumor after re-TUR in their univariate analysis [10], but our multivariate analysis results differ somewhat. Tumor multiplicity and grade and concomitant CIS were revealed as risk factors for residual tumors after re-TURBT in our study, but initial T stage was not a significant predictor of these residual tumors. This difference may be because most of the patients who underwent re-TURBT in this study were at the initial T1 stage.

It was notable that the presence of residual tumors on re-TURBT was associated with higher risks of tumor recurrence compared with patients who had no tumors on re-TURBT. Similar results were reported in a previous study in which tumor-free status at re-TURBT was associated with fewer tumor recurrences and longer times to recurrence [16]. A retrospective study also reported that 83% of patients with residual tumors on re-TURBT developed recurrence compared with 39% of patients at stage T0 [17].

Few studies have analyzed re-TURBT pathology findings as prognosis risk factors; as mentioned above, most studies on re-TURBT in bladder cancer have focused on the stage migration and presence of the residual tumors. In this study, both the tumor pathology and the residual tumor status after re-TURBT were also associated with recurrence; in univariate analysis, stage T1 and high tumor grade in re-TURBT were significantly associated with residual tumor recurrence, and stage T1 in re-TURBT was still statistically significant in multivariate analysis.

Herr et al. reported in their retrospective study that among 710 patients with bladder cancer, including 352 with pT1 tumors, 76% with residual pT1 tumors on restaging TUR progressed to muscle invasion, versus 14% who had no tumor or noninvasive (Ta, CIS) tumors [13]. Moreover, most patients with a persistent T1 tumor on second TUR eventually developed worse disease, even if they responded initially to BCG therapy. Thus, Herr suggested that residual invasive carcinoma (T1) on restaging TURBT might help to identify patients who need immediate radical cystectomy [9, 13]. Our findings are also in line with the results of previous studies. Of 161 patients with initial stage T1, 34 patients had residual T1 tumor, and of these, 27 (79.4%) showed recurrence during follow-up, and eight (23.5%) showed progression above T2.

Our patient population, however, had relatively few patients diagnosed with initial to residual T1 tumors compared with other studies, possibly because in this study, we excluded all incomplete resection patients at the initial TURBT and patients with visible residual tumors on re-TURBT; thus, our proportions of residual T1 may have been low compared with other studies. Nevertheless, in our multivariate analyses, residual stage T1 was a significant factor in disease recurrence and progression. These results agree with Herr et al.’s suggestion of immediate radical cystectomy for T1 patients after re-TURBT.

LVI, the presence of tumor cells in lymphatic vessels and vascular walls, is emerging as a prognostic factor for bladder cancer [18]. Although many studies showed no reliable prognostic value of LVI regarding recurrence and progression outcomes, the results of larger studies show that LVI might be a promising prognostic marker [18]. In the largest multicenter series, which comprised 1,136 patients with high-grade T1 disease treated with radical cystectomy, Fritsche et al. reported that LVI was significantly associated with disease recurrence and overall survival [19]. Furthermore, Kim et al. demonstrated in a meta-analysis that included data from more than 3,900 patients that LVI in TURBT specimens was associated with RFS and PFS in patients with NMIBC [20]. In our study, LVI in initial TURBT specimens was also significantly associated with disease recurrence in univariate and multivariate analyses, as in previous reports. It was also associated with progression in univariate analysis, but the findings were not significant in multivariate analysis.

The fact that local tumor control and accurate tumor staging depend on a complete TUR and reevaluation of the tumor base suggests that a restaging TURBT may be of value in assessing patients with bladder tumors [21, 22]. Re-TURBT reduces the uncertainty of the depth of tumor invasion, better controls the primary tumor, and provides additional pathologic information that may help to select appropriate treatment. In addition, the pathologic findings of re-TURBT itself are also meaningful in predicting the associated disease prognosis, such as recurrence, in NMIBC patients.

There were some limitations to our study. First, we did not include stage T2 among the re-TUR patients in this study, and it could have been meaningful to learn the prognoses of patients who had been diagnosed with NMIBC even after re-TURBT. However, excluding stage T2 patients may have affected our analysis of disease progression risk factors. Second, our study was limited by its retrospective design. Nevertheless, it is meaningful in that we had the largest proportion of patients with NMIBC after re-TURBT compared with other studies excluding Herr. However, there were few prospective studies about re-TURBT findings, and thus, a prospective study design with a larger sample is needed to confirm our presented results.

## Conclusion

In this study, tumor multiplicity, high tumor grade, and concomitant CIS were revealed to be independent risk factors for residual tumor after TURBT. In re-TURBT pathology, T stage is an independent predictor of NMIBC recurrence and progression, and high tumor grade in re-TURBT pathology was also an independent predictor of progression. From the results of previous reports as well as our own experience, re-TURBT is recommended to reduce the chance of residual tumor and to more accurately predict the prognosis in high-risk NMIBC.

## Abbreviations

TURBT: Transurethral resection of bladder tumor
re-TURBT: repeated transurethral resection of bladder tumor
LVI: Lymphovascular invasion
NMIBC: Non muscle invasive bladder cancer
BCG: bacillus Calmette-Gue´rin
CIS: Carcinoma in situ
OR: Odd ratio
HR: Hazard ratio
EAU: European Association of Urology
